# Extraction of Microcrystalline Cellulose and Silica from Agriculture Waste and Its Application in Synthesis of Wheat Gluten and Fish Scales Derived Bioplastic

**DOI:** 10.1155/2022/2297364

**Published:** 2022-08-24

**Authors:** S. Samraj, K. Senthilkumar, P. Induja, M. Venkata Ratnam, G. V. Aatral, G. V. S. Ramakrishna

**Affiliations:** ^1^Department of Chemical Engineering, MVJ College of Engineering, Bangalore 560 067, India; ^2^Department of Chemical Engineering, Kongu Engineering College, Erode, Tamilnadu 638 060, India; ^3^Department of Chemical Engineering, Hindusthan College of Engineering and Technology, Coimbatore, Tamilnadu 641 032, India; ^4^Department of Chemical Engineering, Mettu University, Metu Zuria, Ethiopia; ^5^Department of Food Technology, Kongu Engineering College, Erode, Tamilnadu 638 060, India

## Abstract

Plastics play a significant part in human life and the world we live in. The use of plastics results in detrimental effects on the natural world, which compels us to look for viable replacements. As a result of their enhanced capacity to biodegrade, bioplastics are becoming increasingly important materials. In recent years, there has been a rapid ascent in the utilization of biopolymers in various applications. The objective of this research is to investigate the impact that silica obtained from rice hull ash (RHA) and microcrystalline cellulose (MCC) obtained from groundnut husk have on the properties of bioplastic obtained from wheat gluten and fish scales. The usage of fish scales has been shown to have a positive effect on weight reduction and debasement rates. Microcrystalline cellulose (MCC) is utilized in a wide range of concentrations, and the influence of MCC on bioplastic is researched. The biodegradability tests of bioplastic revealed that the plastic lost 35% of its weight in just 14 days. The experiments that were done to evaluate the chemical stability and tensile strength of the bioplastic indicated that the MCC content has a significant effect in improving the characteristics of the material.

## 1. Introduction

Plastics are now used in almost every aspect of our lives, from human services to home care and from food to design. They have largely replaced traditional materials and made it possible to achieve unprecedented levels of cleanliness and food preservation. It is estimated that only 14% of plastic bundles are collected around the world for subsequent use [1]. It has not taken us long to get to the point where trash made of plastic is polluting both the oceans and the land around them. The challenge that researchers are currently attempting to overcome is the search for appropriate materials that can coordinate plastic execution without having an adverse effect on nature. There are now bioplastics that are available on the market that are distinct from traditional materials [1, 2]. Investigations are being conducted into the creation of potential new bioplastics using a diverse assortment of materials. Bio-based polyethylene terephthalate (PET), which has the potential to replace the petroleum-based PET that is currently used in the manufacturing of drinking bottles. Some types of beverage bottles, for instance, are fabricated using bio-based PET material that is derived from sugar sticks and biodegradable polymers that are derived from oil [2].

According to some accounts, parkesine was the first man-made plastic. Since then, numerous advancements have been made, and it has evolved into a substance that is commonplace in our everyday lives. After that, it was discovered that it had significant effects on the environment, which made it necessary to look for chemicals that would not harm the environment. Everyone was interested in bioplastic due to the fact that it was good for the environment and quickly broke down [3, 4]. The production of bioplastics from plant matter and agricultural byproducts has been the subject of a number of studies that have been carried out [3–5]. Tea debris, agricultural waste, eggshells, wheat gluten, and feathers from chickens are some of the materials that are used in the production of bioplastic. In some studies [3–5], wheat gluten was used to create a bioplastic. Wheat gluten is a plant protein that is distinct from other plant proteins in a number of ways, including the fact that it is simple to obtain, that it decomposes rapidly, that it is inexpensive, that it possesses one-of-a-kind viscoelastic properties, and that it can cross-link when heated. A number of studies on the development of biopolymer-based food packaging have been prompted by the nonbiodegradability of plastics and the potential transfer of hazardous substances from plastics to food [6]. These two factors have led to the need for these studies. Proteins, particularly those of animal origin, are the biomaterials that are used the most frequently in the food industry. Furthermore, the inherent properties of proteins, such as their structure and molecular weight, amino acid composition, chemical interactions, and chemical environmental conditions such as temperature and pH, favour the production of nanostructures that are aimed at increasing bioactive availability [7, 8]. Wheat gluten is one example of this type of renewable material. It has a high protein content, excellent biodegradability, fantastic thermoplastic properties, and simpler removal processing, all of which make it ideal for use in agricultural and bundling applications [9, 10]. In addition, the utilization of proteins as raw materials promotes the formation of a complicated network of polymers by making a wide variety of chemical functionalities accessible to a wide variety of amino acids [11]. This is the case because proteins contain an abundance of amino acids. As a result of the uncertainty regarding the presence of rice husk ash (RHA) silica, an extraction method that requires a lower temperature and uses less energy can now be used to separate goods.

RHA is currently utilized in the manufacturing of products that are derived from silicone, including silicon carbide, nitride, and tetrachloride [12]. The process involved dissolving silica with a soluble base at a temperature of 100°C in order to produce sodium silicate and, as a result, silica aqua gel by adding corrosive hydrochloride in order to lower the pH to 7.0. In order to make silica oxy-gel, the silica aqua gel needed to be washed for 24 hrs at 80°C and subsequently rinsed twice [13]. Bundling materials absolutely cannot be constructed without the incorporation of microcrystalline cellulose, also known as MCC. In order to produce cellulose MCC, cellulose is reacted with a liquid arrangement of a solid mineral corrosive at a bubbling temperature for an extended period of time. This process continues until the level-off DP of cellulose is reached. MCC has high compressibility and, when added in minute amounts, it increases the other diluents' potential for being compressed further. It has a level of smoothness that makes it possible to crush it, and it has crumbling properties [14]. At the moment, particular strands that are made from nonwood fibres are created in order to be used for bundling and the production of paper. The ongoing expansion helps accomplish two major goals in this context. In several rapidly developing nations across Asia, Africa, and Latin America, there has been an immediate reduction in the availability of wood, which has been accompanied by an increase in interest in advertisements for mash. In addition, agricultural deposits are produced in large quantities; however, they are routinely discarded as a waste of excellent materials that can serve as a substitute for wood due to the fact that they are readily available everywhere and can be utilized effectively. Utilizing rural deposits has many advantages, including positive effects on the economy, conditions, and creative output [15–18]. Rural deposits are abundant and cannot be depleted. The cellulose that is used to make microcrystalline cellulose is hydrolyzed under controlled conditions to produce the material. Cellulose can be obtained from a variety of sources, including the maturation of microorganisms or plants [19, 20].

There are several instances of bioplastics produced through MCC extraction in the scientific literature. The current study focused on material combinations that have not been published before. The tensile strength of epoxy resins can be increased by utilising fish scales, which are a less expensive material. However, little study has been conducted on the use of fish scales as a bio filler in the manufacturing of composites including wheat gluten and MCC. The primary objective of this research is to extract microcrystalline cellulose from groundnut husk in order to investigate the characteristics and qualities that it possesses as a diluent in comparison to the standard cellulose used in the industry. In addition, an attempt was made to evaluate the effect of silica produced from RHA and MCC obtained from groundnut husk on bioplastic derived from wheat gluten and fish scales. This evaluation was carried out in an attempt to determine how these two substances interact with the bioplastic. A number of investigations, including tensile strength testing, degradability testing, and compound testing [21, 22], as well as characterization studies utilising SEM, XRD, and FTIR, have been carried out.

## 2. Experimental

### 2.1. Chemicals and Raw Materials

All synthetic chemicals utilized were of analytical reagent grade. Hydrochloric acid and sodium hydroxide were utilized to alter the pH. Distilled water was utilized all through the exploratory examinations. Raw materials used in this research include rice hull ash, groundnut husk, wheat, and fish scales obtained locally in Tamil Nadu State of India.

### 2.2. Preparation of Silica from Rice Hull Ash

**Figure 1:**
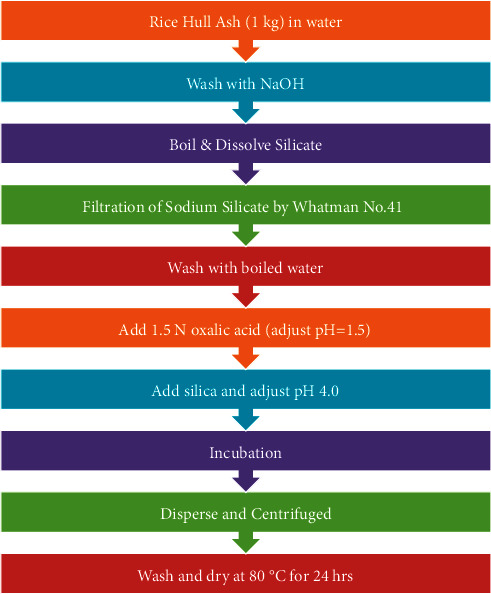
Flow diagram for extraction of silica from RHA.

The sequential description of the process of extraction of silica from RHA is presented in Figure 1. The rice frame was obtained from a rice factory in the vicinity. The rice body was then finished and the RHA was collected. 1 kg of RHA was treated with 5 L of 1 N NaOH and then boiled to disintegrate silica and deliver sodium silicate. The solution was filtered by Whatman No. 41 filter paper, and the carbon accumulation was washed with bubbling water and then allowed to cool at room temperature. The acid solution was formed by adding 1.5 N of oxalic acid to 100 ml of deionized water until pH 1.5 was reached. A silica solution is added to an acid solution until a pH of 4 is reached. Then it was hatched to advance the production of silica gel. The silica gel produced was squashed in measuring glasses, dispersed in water, and then centrifuged to extract solvent salts. It was washed and dried at 80°C for 24 hrs [12].

### 2.3. Preparation of MCC from Groundnut Husk

**Figure 2:**
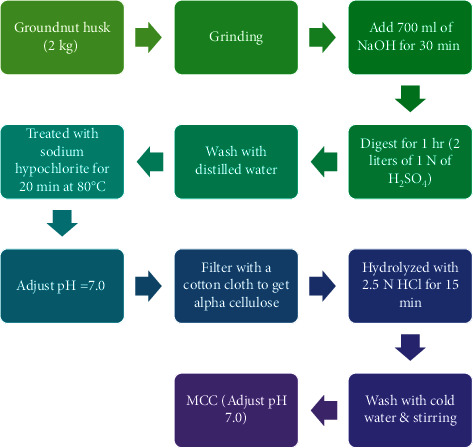
Flow diagram for extraction of MCC from groundnut.

The groundnut husk (2 kg) was sundried for 7 days before being coarsely milled into powder for 30 min. 1.5 kg of groundnut husk powder was delignified with 700 ml of NaOH (5% w/v). The slurry was filtered through a cotton cloth before being treated with 2 litres of 1 N H_2_SO_4_ and digested for 1 hr. The resulting slurry was treated with distilled water and rinsed. It was also bleached for 20 min at 8°C with a sodium hypochlorite solution, then rinsed with water until it became neutral. The water content was manually squeezed out after filtering using a cotton towel, and it was then dried at 60°C for 6 hrs [15]. The technique previously described was used with a minor modification to get alpha-cellulose hydrolyzed with 2.5 N HCl for 15 min [15]. At that point, the mixture was rinsed with cold water, vigorously mixed, and set aside for the time being until the MCC was washed with water to achieve a neutral state, after which it was pressed and dried at 60°C for 1 hr. It was finally ground into a fine powder [20].  Figure 2 presents the steps involved in the extraction of MCC from groundnuts.

### 2.4. Preparation of Bioplastic

**Figure 3:**
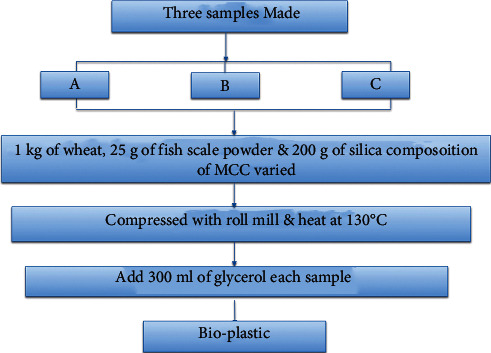
Flow diagram for the production of bioplastics.

Wheat was collected from a nearby market, washed and sundried for three days, and then ground into a fine powder. Wheat gluten was washed with water several times until no white colour water was recovered. It was then powdered and dried [9]. Fish scales were purchased at a local market, cleaned with water several times, dried, and ground into powder [6, 7]. Finally, three samples were created, each containing 1 kg of wheat gluten, 25 mg of fish scale powder, and 200 g of silica. Samples A, B, and C contained 200 g, 300 g, and 400 g of MCC, respectively, and these combinations were properly mixed. 300 ml of glycerol was added to each sample before being crushed in a roll mill and heated to 130°C before being stored in an airtight container.  Figure 3 depicts an overview of the bioplastic production process.

### 2.5. Characterization of Bioplastics

The bioplastics acquired were described and tried in biodegradation and chemical tests for weight reduction and tensile strength.

#### 2.5.1. Biodegradation Test

Test samples (5 g) were vacuum dried at 450°C for 24 hrs, properly weighed, and then submerged in the strong waste blend. They were then tested for biodegradability. The waste blend is a combination of leaves, paper garbage, and bovine dung, as well as food waste, soil treatment seeds, urea, wood debris, and water. The combination was put in a broiler at 55°C, where thermophilic microorganisms thrived. Carbon can be mineralized towards the end of biodegradable polymers after disposal due to biodegradation. The samples were weighted at regular intervals, and the amount of weight loss was calculated using the equation:

(1) $$\begin{array}{c} \%\text{ weight loss}=\frac{W_{1}-W_{o}}{W_{1}}\times 100, \end{array}$$

where *W*_1_ is the dry weight before degradation and *W*_o_ is the dry weight at time *t*.

#### 2.5.2. Chemical Tests

*(1) Effect of Acid*. Test samples (5 g) were precisely measured and then immersed in sulfuric acid with concentrations of 10%, 20%, and 30%. The samples were dried and weighed periodically in order to determine the percentage of weight loss after each time period. With the objective of making a comparison between the produced bioplastic and the other known types of plastic, a sample of polystyrene was exposed to the same test for 30% concentration of sulfuric acid [12].

*(2) Effect of Alkalis*. Test samples (5 g) were accurately measured and then placed in a soluble base arrangement (sodium hydroxide) with concentrations of 10%, 20%, and 30%. The samples were dried and weighed periodically for 10 days in order to determine the percentage of weight loss after each time period. With the objective of making a comparison, sample of polystyrene was exposed to the same test by using NaOH (30% concentration) [12].

#### 2.5.3. Tensile Test

Tensile tests were performed using Shimadzu AG-IS testing equipment with a 1 KN load-cell in accordance with ASTM D638-10 Standard Test Method for Tensile Properties of Plastics. Young's modulus, stiffness, and % elongation were found with an initial gauge separation of 100 mm and a crosshead speed of 50 mm/min.

## 3. Results and Discussion

### 3.1. Extraction of Silica and MCC

The yield given by the extraction strategy suggests that this approach may have an impact on the MCC produced; the best yield of the MCC was obtained by the maceration strategy. The influence of MCC content on the elasticity of a wheat gluten-based bioplastic was studied. The silica will be disintegrated from the RHA obtained when treated with NaOH. The silica extraction from RHA is determined to be 68.7%. The MCC recovered from the groundnut husk yield is 14.2%. The fish scales which have a minimum or not much commercial worth might be exploited as biodegradable reinforcement.

### 3.2. The Fish Scale Characterization

**Figure 4:**
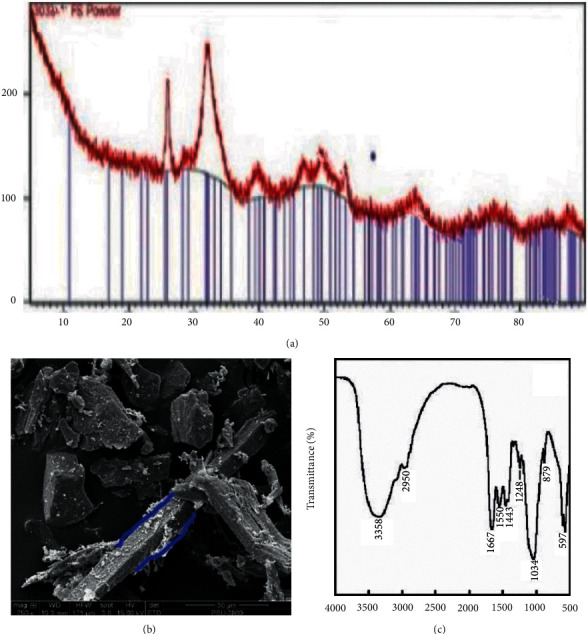
(a) XRD pattern, (b) SEM micrograph, (c) FTIR spectrum of the fish scale powder.

The X-ray diffraction analysis (XRD) of the fish scale sample was conducted to analyze the crystallinity (Figure 4(a)). The XRD of the fish scale powder was identified to be a hydroxyapatite structure (PDF#01-086-0740; hydroxyapatite; calcium phosphate hydroxide, Ca_5_ (PO_4_)_3_(OH)). From the XRD, it is evident from the two peaks at 2*Ɵ* values of 27° and 33° that the sample is crystalline in nature.The surface morphology of the fish scale powder examination was carried out utilising SEM. The fish scale powder sample was coated with gold in a plasma sputtering apparatus.  Figure 4(b) demonstrates that the SEM image of fish scale powder on a 50 *μ*m scale with 750× magnification and it exhibits a rakish, bar-like, and unpredictable shape with a size of 168 *μ*m. The FTIR spectra is represented in Figure 4(c). The wavenumbers at around 600 and 1000 cm^−1^ compared to the carbonate anions that were filled in with phosphate particles in the apatite grid. The FTIR image of Figure 4(c) also indicates the -OH stretching at 3358 cm^−1^, symmetric -CH_2_ stretching at 2950 cm^−1^, stretching of C=O at 1667 cm^−1^, bending of CH_2_ at 1443 cm^−1^, and stretching of C-O at 1034 cm^−1^.

### 3.3. Characterization of Bioplastics

The results of the biodegradation test showed that samples A, B, and C lost 5%, 6%, and 8%, respectively, in the first three days and 32%, 34%, and 35%, respectively, after 14 days. The findings suggested that the weight loss of the produced bioplastic increased by increasing the concentration of sulfuric acid from 10% to 20%, and then, the weight loss of the bioplastic became reduced at a 30% sulfuric acid concentration. These results can be explained by the fact that, by increasing the acid concentration from 10% to 20%, the acid content increased and, hence, the weight loss increased. However, at 30% acid concentration, there was a reduction in water content, which promoted the ponding rupture by acids. The weight loss of the bioplastic at 30% acid content is less in comparison with the standard used for comparison, i.e., polystyrene (30%). As the concentration of NaOH increases, the weight loss increases. The total weight loss of samples A, B, and C was 54%, 57%, and 58%, respectively, after 10 days for 30% NaOH. Compared to polystyrene, which lost 48% of its weight when exposed to alkalis, the bioplastic that was made did not stand up well to alkalis.

The inclusion of MCC has an effect on the bioplastic's tensile strength. The addition of MCC increased stiffness from 6 MPa to 14 MPa, which was attributed to the natural adhesion of the MCC-starch contact due to the chemical similarities between RHA and MCC. In contrast, when MCC concentration increased the elongation was reduced. When the MCC content was increased from 200 g to 300 g, the stiffness of the bioplastic increased (0.45 GPa to 1.15 GPa). Sample B was determined to be the stiffest, with 300 g of MCC and almost double the elasticity of sample C (0.6 GPa).

## 4. Conclusions

Bioplastics are not only made from sustainable biomass sources, but they may also be manufactured from the byproducts of agricultural processes. In comparison to traditional plastics, bioplastics are responsible for around an 80% decrease in greenhouse gas emissions and have the potential to extend the shelf life of food. After 14 days, the samples had lost a total of 32%, 34%, and 35% of their original weight, according to the findings of the biodegradation test. At an acid concentration of 30%, there was a reduction in the water content, which accelerated the acid burst ponding. After ten days, the samples A, B, and C saw a maximum weight loss of 44%, 57%, and 58%, respectively. The bioplastic was subjected to tensile testing, which was analysed. Sample B, which included 300 g of MCC, was the one that was determined to have the maximum stiffness.

## Data Availability

All the data are available in the manuscript.
